# Protective and therapeutic role of melatonin against tunicamycin-induced ER stress in testicular tissue of rats

**DOI:** 10.22038/IJBMS.2022.58719.13043

**Published:** 2022-02

**Authors:** Musa Tatar, Ülker Eren

**Affiliations:** 1 Department of Histology and Embryology, Faculty of Veterinary Medicine, Kastamonu University, Kastamonu, Turkey; 2 Department of Histology and Embryology, Faculty of Veterinary Medicine, Aydın Adnan Menderes University, Aydin, Turkey

**Keywords:** Apoptosis, Endoplasmic reticulum – stress, Endoplasmic reticulum - chaperone BiP, Melatonin, Testis, Tunicamycin

## Abstract

**Objective(s):**

This study aimed to investigate the possible consequences of administering exogenous melatonin as prevention or treatment against tunicamycin-induced endoplasmic reticulum (ER) stress in the testicular tissue of rats.

**Materials and Methods:**

In this study, 42 adult Sprague Dawley rats, randomly divided into seven equal groups, were administered intraperitoneal tunicamycin to induce ER stress. Both prophylactic (PMel) and therapeutic melatonin (TMel) groups were administered melatonin for seven days. ER stress in the cell was detected through immunohistochemical and molecular analyses using GPR78 expression.

**Results:**

Increased oxidant levels and apoptosis rates were shown in testicular tissue because of ER stress. The sections in the melatonin-administered and control groups were similar, with melatonin-administered groups showing an increase in the antioxidant ratio. Histometric examinations revealed both TMel and melatonin applications reduced the diameter of the tubules. However, immunohistochemical and molecular analyses showed that PMel administration decreased the concentration of GRP78 more effectively than TMel.

**Conclusion:**

Applying melatonin prior to cell damage occurrence can be recommended for its effectiveness in protecting from tunicamycin-induced ER stress.

## Introduction

The folding stage of proteins occurs through complex processes when the probability of error is very high. Many factors such as N-linked glycosylation inhibition, hypoxia, impaired Ca^ 2+^ homeostasis, infections, oxidative stress, and temperature can affect the correct folding of proteins (1). These factors that cause impaired endoplasmic reticulum (ER) function and accumulation of unfolded or misfolded proteins in the ER lumen also trigger cellular ER stress. The cells activate the unfolded protein response (UPR) pathway to prevent ER stress and to restore ER homeostasis (2). Oxidative stress-mediated ER stress in testicular tissue is known to occur in response to exposure to various toxic agents. Excessive production of reactive oxygen species (ROS) has been linked to ER stress and UPR (3, 4). Additionally, studies have shown that antioxidants attenuate oxidative stress-mediated ER stress and activation of the UPR signal (5). Tunicamycin (TM) has been used to induce experimental ER stress in cell cultures or animals as it inhibits the formation of N-linked glycoproteins by inhibiting the N-acetylglucosamine transferases (6, 7). Melatonin (N-acetyl-5-methoxytryptomine), the main secretory product of the pineal gland, functions as a powerful antioxidant and free radical scavenger (8). Melatonin has been reported to prevent many diseases associated with ER stress and to repair cell damage caused by ER stress (9, 10). ER stress contributes to the pathogenesis of many diseases. Neurodegenerative diseases (e.g., amyotrophic lateral sclerosis, Parkinson’s, Alzheimer’s, and Huntington’s), diseases with ischemia/reperfusion damage, metabolic diseases (e.g., Type 2 diabetes and obesity), and cancer are among the diseases that have been mentioned (11, 12). Due to this close physiological relationship between the structure and function of ER, disorders in ER structure have been linked to diseases (13). The ER environment can be regulated using a variety of factors with melatonin gaining increasing attention in this regard (14). Therefore, this study aimed to investigate the protective and therapeutic effects of melatonin against tunicamycin-induced ER stress in rat testis in terms of histological, immunohistochemical, molecular biological, biochemical, and enzyme histochemistry. Thus, by revealing the effect that ER stress has on testicular seminiferous tubular cells, this study plans to contribute to male infertility and development of new treatment approaches to be applied in this regard. 

## Materials and Methods

***Animals and treatments***

**Figure 1:**
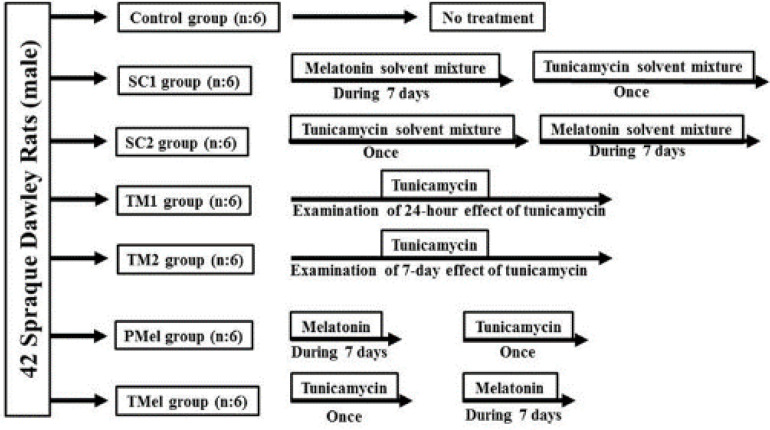
Flowchart of the study. Tunicamycin-solvent mixture (150 mM dextrose with 1% DMSO); Melatonin solvent mixture (Phosphate buffer saline [PBS] containing a 1% concentration of ethanol); SC1 = Vehicle control-1; SC2 = Vehicle control-2; TM1 = Tunicamycin1; TM2 = Tunicamycin2; PMel = Profoloctic melatonin; TMel = Treatment melatonin

This study was conducted with the approval of the Ethics Committee of Burdur Mehmet Akif Ersoy University (Ethics committee number: 2017/282). Forty-two adult male Sprague Dawley rats (approximately 90 days old) were used in this study. The rats were provided by the Burdur Mehmet Akif Ersoy University Experimental Animal Production and Experimental Research Center. The animals were housed in polycarbonate rat cages (3 rats per cage) at 24 °C ± 1 °C, 50-55% humidity, and a 14 hr light/10 hr dark cycle. A laboratory rodent diet (provided by xxx) and water were supplied *ad libitum* during the experimental period. The rats were randomly separated into seven groups consisting of six animals each. TM was used to create acute ER stress as previously described (7, 15). The control group received no treatment. TM, melatonin, and solvent applications were administered intraperitoneally to the other groups. A tunicamycin-solvent mixture (150 mM dextrose with 1% DMSO) was injected once to the sham control 1 (SC 1) group following the melatonin solvent mixture (Phosphate buffer saline [PBS] containing a 1% concentration of ethanol) application for 7 days. In the sham control 2 (SC 2) group, the tunicamycin-solvent mixture followed by the melatonin-solvent mixture was applied for 7 days. A single dose of 200 µg/kg TM was applied to the one-day effect group for tunicamycin (TM 1) to examine the 24-hour effect of TM. The tunicamycin 1-week effect group (TM 2) was given a single dose of 200 µg/kg TM over seven days to examine the seven-day effect of TM. The prophylactic melatonin group (PMel) was administered a single dose of 200 µg/kg TM followed by a melatonin dose of 20 mg/kg (16) for seven days. In the melatonin-treatment group (TMel), day 1 started with a single dose of 200 µg/kg TM administration, then day 2 involved administering melatonin daily at a dose of 20 mg/kg for seven days (Figure 1). In order to reduce circadian rhythm effects, all drug applications and euthanasia procedures were performed between 9:00 and 11:00 AM (17).

Sample collection and preparation

The rats were made to fast overnight, and the experiment was completed by recording the body weights in the morning. The rats were administered xylazine-ketamine (10 mg/kg-90 mg/kg, IP) as an anesthetic followed by euthanasia using the cervical dislocation technique. The testes were dissected, and each testis was weighed individually. The tissue from the left testis was frozen at –80 °C until the experimental stage to measure the density of MDA, SOD, and GRP78 (18). The right testes were fixed in Bouin’s solution, after which the samples were passed through a gradational series of alcohols, methyl benzoate, and benzole followed by embedding in paraplast (19). Cross-sections (6 µm thickness) were taken from the paraffin blocks at 100 µm spaces. Slides were stained with Crossman’s triple stain to determine the histological and histometric changes (20).

Histological changes

Histological changes such as tubular atrophy, exfoliation of germ cells, and tubular or sub-basal vacuolization in the lumen were examined in two sections taken from each animal (21).

Histomorphometry

A total of six serial sections from each rat were stained in the study. The seminiferous tubule diameters (STD) and seminiferous epithelium heights (SEH) were measured in 20 selected round or nearly round tubules in VII-VIII and XII-XIV stages in each section (22). The sections were examined with the help of an image analysis program (CellSens standard program, Olympus) attached to a microscope (BX51, Olympus), and the slides were viewed with a digital camera (DP74, Olympus). In each section 150 tubules were analyzed, and the percent ratio of the Stage XIV tubules was registered (23).

Immunohistochemistry of GRP78

The expression of GRP78 was observed using a rabbit anti-GRP78 polyclonal antibody (Abcam ab21685) through the streptavidin-biotin complex staining method in three slides from each animal (24). The samples were dewaxed and kept in graded alcohol. Then, the sections were transferred to citrate buffer (pH 6.0) for antigen retrieval and boiled for 30 min. Afterward, the slides were treated with a 3% H_2_O_2_ distilled water solution for 15 min to stop endogenous peroxidase activity. The slides were placed in a blocking solution for 30 min (Histostain-Plus IHC Kit, HRP, broad-spectrum, 859043). After this process, the sections were incubated for three hours at 37 °C with a primary antibody diluted to 1:200 (Abcam ab21685). Negative control sections were processed using tris-buffered saline (TBS, pH 7.4) instead of the primary antibody. The sections were incubated in biotinylated secondary antibody for 30 min, incubated in Streptavidin–HRP (horseradish peroxidase), and processed with 3,3-diaminobenzidine (DAB) for two minutes. Finally, all sections were counterstained with Harris’s Hematoxylin and closed using entellan. The stained sections were examined through a microscope (BX51, Olympus), and the slides were viewed with a camera (DP74, Olympus). Immunostaining intensity was evaluated subjectively as 0-negative, 1-low, 2-poor, 3-moderate, or 4-high (25). Twenty tubules were selected for each section, and the density of brown precipitation as GRP78-positive cells was evaluated.

Protein extraction and western blot

Western blotting was conducted as previously described by Kurien and Scofield (26). Freshly collected tissue samples were instantly transferred to liquid nitrogen and kept at −80 °C until the western blot was performed. The testis tissue was homogenized using the RIPA lysis buffer (Santa Cruz Biotechnology, USA) with a protease inhibitor cocktail. After being centrifuged at 13,000 × g and 4 °C for 15 min, the supernatant was collected. Protein concentration in the supernatant was determined suitable for use by the SMART Micro BCA protein assay kit (Intron Biotechnology). Proteins loaded onto 10% SDS-PAGE polyacrylamide gel in equal amounts for each group were separated in the electrophoresis system and then transferred to a membrane. After transfer, the membrane was blocked in 5% skimmed milk at room temperature for 1 hr. The blots were incubated with the primary antibody (rabbit Anti-GRP78 polyclonal antibody, Abcam ab21685, 1:1000 dilution; mouse Anti-β-actin monoclonal antibody, Santa Cruz sc-47778, 1:1000 dilution) overnight at 4 °C. The blots were then washed and incubated with horseradish peroxidase (HRP)-conjugated secondary antibodies (sc-2004; sc-2005, polyclonal, Santa Cruz Biotechnology) for 1 hr at room temperature. Immunoreactive bands were detected by a chemiluminescence substrate using ECL Reagent (K820-50, BioVision). β-Actin was used for normalizing it, and band intensities were quantified using UVP Life Science software (Cambridge, UK).

***Measurement of SOD and MDA status levels in testes***

***Testis tissue s*** amples were homogenized in PBS solution, using a homogenizer at 2000 rpm for 1 min. Tissue homogenates were centrifuged at 11,300 rpm and 4 °C for 10 min. The supernatants were collected and analyzed. To calculate the oxidant and anti-oxidant parameters in the supernatants, the total protein was calculated as mg/ml protein using the Biuret method. SOD activity was calculated at 560 nm with respect to Sun *et al*. method (27) and expressed as U/mg of tissue protein. The MDA levels of the tissues were determined using the method previously reported by Ohkawa *et al*. (28). Briefly, the color intensity of MDA, a colored complex molecule formed by thiobarbituric acid incubation, was calculated using spectrophotometric measurement at 532 nm. The obtained results were expressed as nmol/mg of protein.

Measurement of apoptosis using the terminal deoxynucleotidyl transferase dUTP nick end labeling (TUNEL) assay

The TUNEL method was carried out using an *in situ* cell death detection kit (cat 11684817910) from two slides of each animal (29, 30) following the manufacturer’s protocols. The TUNEL positive germ cells were investigated in 100 seminiferous tubules/sections. In the evaluated tubules, the mean number of TUNEL-positive cells per tubule was expressed as apoptotic index and percentage of tubules with apoptotic cells (TWAC%) (31, 32).

Statistical analysis

The SPSS 22.0 program package was used to perform statistical analysis. The results were expressed as mean ± standard deviation (SD). All the traits were controlled for normal distribution using Kolmogorov-Smirnov and Shapiro-Wilk tests. The values were analyzed based on groups using the Kruskal-Wallis test or one-way analysis of variance (ANOVA) depending on whether or not the data were distributed normally. Differences between groups were evaluated using Duncan’s *post-hoc* test. Chi-square analysis was applied for the values determined by counting. Statistical significance was determined at *P*<0.05 for all evaluations.

## Results

***Body and testis weight***

The body and testicular weights did not vary between groups at the beginning and the end of the experiment (Table 1).

***Histological appearance and histomorphometry***

**Figure 2:**
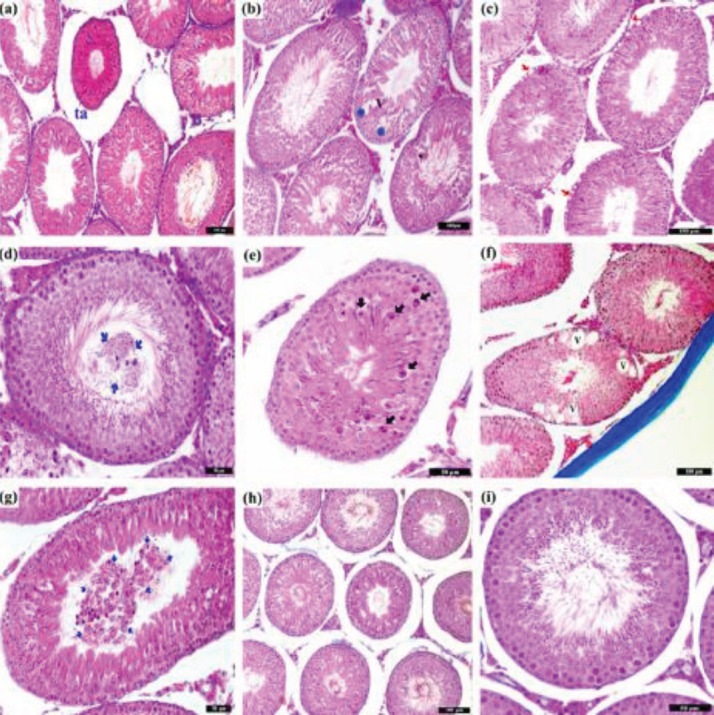
Tubule images in the testicular tissue from the TM1 group (a, b, c, d), TM2 group (e, f, g), and PMel group (h, i) using the Triple Staining Method. ta = tubular atrophy, V = Vacuole. Blue asterisks show shedding of germ cells, red arrows show sub-basal vacuolization, blue arrows mark exfoliation and black arrows show germ cell degeneration

The testicular tissue looked similar among control, SC1, and SC2 groups. Although the appearance in the experimental groups was similar to the control groups in general, tubules with histological changes were observed. The degenerative changes appeared more in the groups treated with TM than in the melatonin applied groups. In the TM1 group, decreases in seminiferous tubule diameter (tubular atrophy), epithelial layers, and sub-basal vacuolization, as well as exfoliation of germ cells into the lumen of the tubule, were observed (Figures 2a, 2d). The TM2 group was detected to have germ cells in degenerated form through numerous eosinophilic cytoplasms and nuclear condensation, larger and more vacuolization, and shedding of germ cells into the tubular lumen (Figures 2e, 2g). Although seminiferous tubules were found with degenerative changes in TM1 and TM2 groups, no significant change had occurred in their Sertoli and Leydig cell morphologies. Tubular structures in PMel and TMel groups seemed more like the control group. However, the decrease in tubular diameter compared with the control group was quite remarkable (Figures 2h, 2i). Degenerative changes were also observed in some tubules in the PMel group. Fewer degenerative cells were detected in this group compared with the tunicamycin groups. While epithelial vacuolization was observed in the TMel group, the degenerative cells were not found to be different.

STD and SEH values from Stages VII-VIII and XII-XIV for control, SC1, SC2, and experimental groups were measured (Table 2). The STDs in TM1, TM2, PMel, and TMel groups at stages VII–VIII were lower compared with control, SC1, and SC2 groups (*P*<0.001). The lowest STD at stages VII-VIII was found in the PMel group (*P*<0.001). The STDs in the melatonin treatment groups were lower compared with the TM2 group (see Table 2). SEH in the PMel group was lowest at stages VII-VIII (*P*<0.001). The highest STD at stages XII-XIV was found in control and SC1 groups (*P*<0.001). The lowest STD at stages XII-XIV was noted in the PMel group (*P*<0.001). SEH at stages XII-XIV was similar to TMel and SC1 groups (*P*>0.05) and was significantly higher than in the other groups (*P*<0.001). TM and melatonin administration reduced the percentage rates of the stage XIV tubules compared with control, SC1, and SC2 groups, but these decreases were not significant (*P*>0.05; Table 2).

***Immunohistochemistry***

**Figure 3:**
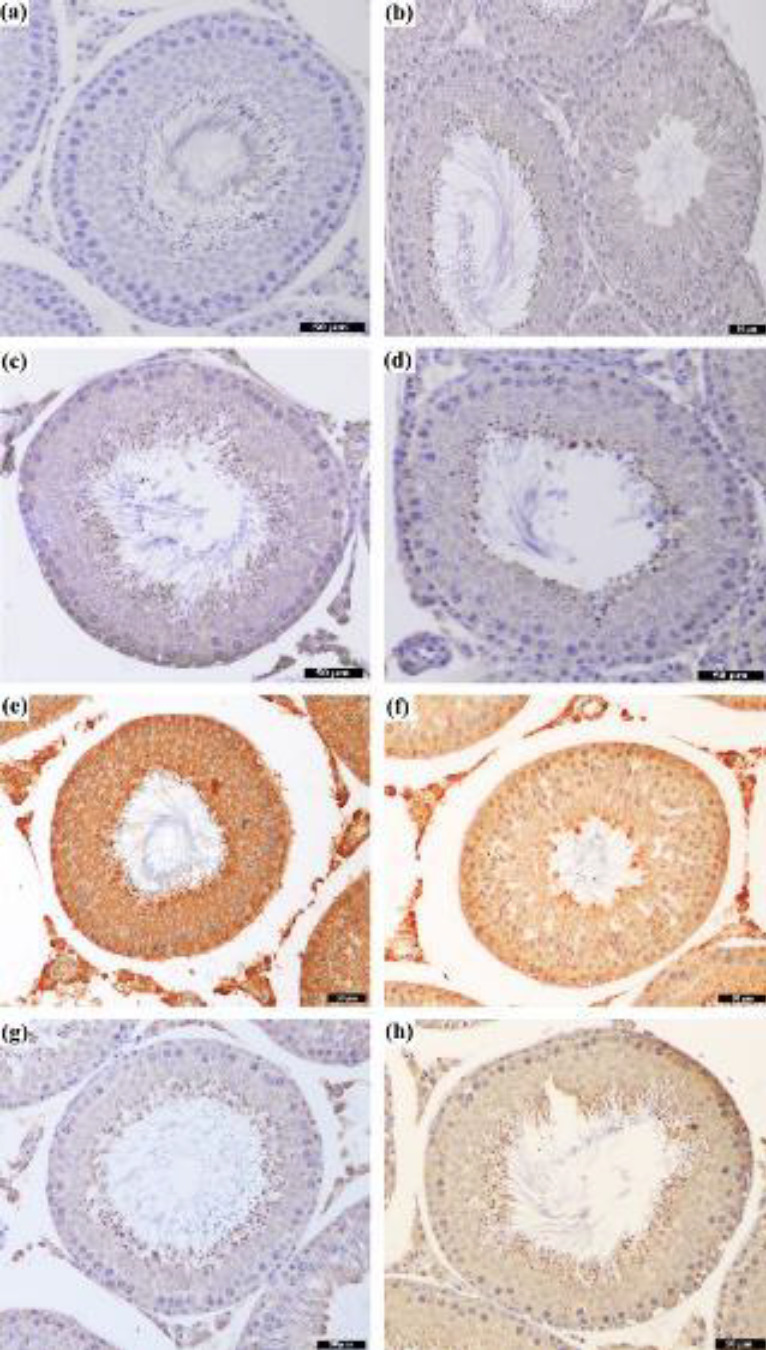
The appearance of GRP78 expression in the seminiferous tubule of the rat testis. (a) Negative control group, (b) Control group, (c) SC1 group, (d) SC2 group, (e) TM1 group, (f) TM2 group, (g) PMel group, and (h) TMel group using the streptavidin-biotin complex (sABC) staining method

**Figure 4:**
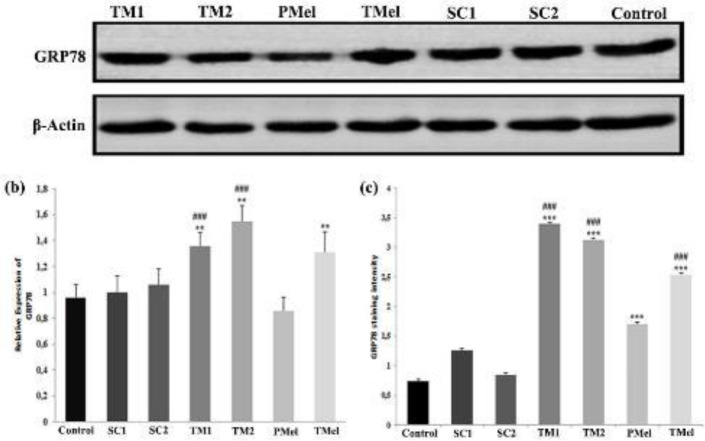
Western blot (a, b) and immunohistochemistry (c) series of GRP78 expression levels analyses in rat testis. Relative band density of GRP78 was quantified and normalized to the β-actin expression. Data were normalized by dividing arbitrary units of GRP78-β-actin. ** *P*<0.003 versus control group; *** *P*<0.001 versus control group; ###*P*<0.001 versus PMel group. Data are expressed as the means ± SD. n = 6 rats for each group

GRP78 positivity was measured in all groups in the tubular epithelial cells and interstitial cells, but the staining intensities varied in all groups. No brown precipitation was observed in the negative control (Figure 3a). GRP78 positivity was most intensely observed in TM1 and TM2 groups (Figures 3e, 3f). PMel and TMel groups had less intense staining than the tunicamycin administered groups (Figures 3g, 3h). The GRP78 staining intensity of the cells in the seminiferous tubules and interstitial area in the sections belonging to control, SC1, SC2, and experimental groups was scored subjectively. When examining TM1, TM2, and TMel groups, the staining intensity was greater compared with the other groups (*P*<0.001). The most intense staining was observed in the TM1 group. The intensity of staining was significantly lower in the PMel group compared with that in TM1, TM2, and TMel groups (*P*<0.001; Figure 4c).

Determining the GRP78 protein using western blotting

The expression levels of GRP78 in the testicular tissue samples were measured using the western blot method (Figures 4a, 4b). The expression of GRP78 in the TM2 group was greater compared with control, SC1, SC2, and PMel groups (*P*<0.005). The GRP78 expression levels in the TMel group were not different compared with TM1 and TM2 groups (*P*>0.05). The GRP78 expression level in the PMel group was lower than that in TM1, TM2, and TMel groups (*P*<0.005). In addition, the relative fold changes in protein levels for GRP78 (protein/β-actin) were compared based on the control group. The relative fold changes were found to have a 1.04-, 1.1-, 1.41-, 1.61-, and 1.36-fold increase in SC1, SC2, TM1, TM2, and TMel groups, respectively, and a 1.1-fold decrease in the PMel group.

***MDA and SOD levels in testis tissue***

MDA and SOD levels determined in testicular tissues are shown in Figures 6a and 6b. Tunicamycin-administered groups had significantly higher MDA levels compared with control, SC1, and SC2 groups (*P*<0.001). No significant difference existed in MDA activity of control, SC1, or SC2 groups (*P*>0.05). The MDA levels in the PMel group were lower than in all other groups (*P*<0.001). The SOD levels were higher in the melatonin-administered groups, with the highest value in the PMel group (*P*<0.001). The lowest SOD value was noted in the TM1 group, with the difference compared with the melatonin groups also being significant (*P*<0.001).

***Evaluation of germ cell apoptosis***

**Figure 5:**
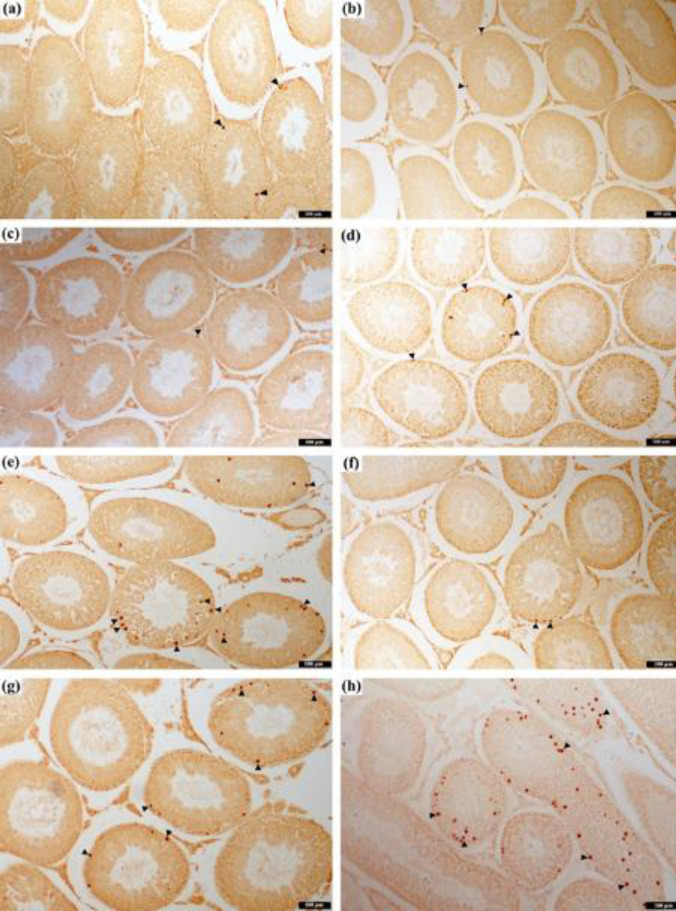
Appearance of apoptosis in the seminiferous tubules using the TUNEL staining technique. (a) Control group, (b) SC1 group, (c) SC2 group, (d) PMel group, (f) TMel group, (e & g) TM1 group, (h) TM2 group. Black arrows indicate TUNEL-positive germ cells

**Figure 6:**
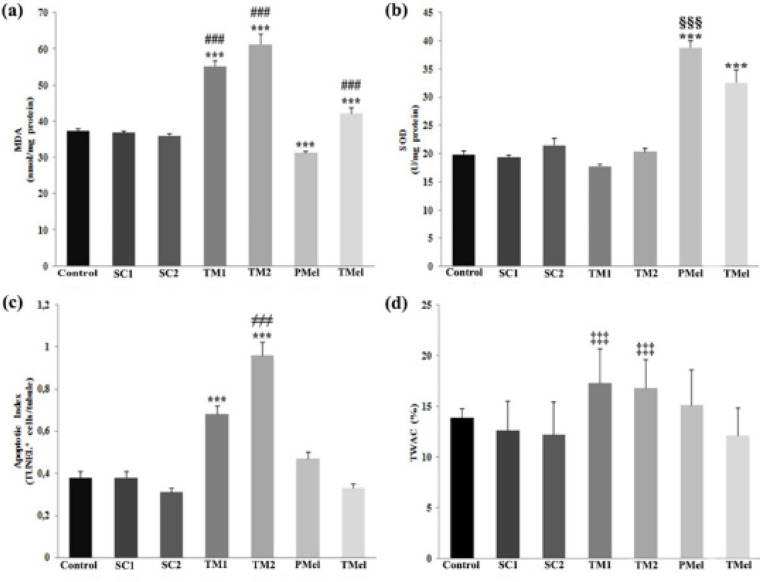
MDA (a) and SOD (b) levels, and Apoptotic index (c) and TWAC (d) analyses of the rat testicular tissues. *** *P*<0.001 versus control group; ###*P*<0.001 versus PMel group; §§§P<0.001 versus TMel group; ≠≠≠ *P*<0.001 versus TM1 group; ‡‡‡ *P*<0.001 versus SC1 and SC2 groups. Data are expressed as the means ± SD. n = 6 rats for each group

The average number of TUNEL positive germ cells per tubule (apoptotic index) and the percentage of tubules with apoptotic cells (TWAC%) are given in Figures 6c and 6d, respectively. Although apoptotic cells were detected in the seminiferous tubules in all groups (Figure 5), the apoptotic index was greater in TM1 (Figures 5e, 5g) and TM2 (Figure 5h) groups compared with other groups (*P*<0.001). The greatest number of TUNEL positive germ cells was detected in the TM2 group. No difference was noted among TMel, control, SC1, and SC2 groups in terms of the apoptotic index (*P*>0.05; Figures 6c, 6d). In addition, fewer TUNEL positive germ cells were observed in the TMel group than in the PMel group (*P*<0.001). TM1 and TM2 groups had the greatest TWAC%. As a result, both TWAC% and apoptotic index were significantly lower in the TMel group compared with the other groups.

**Table 1:** Bodyweight values at the beginning and end of rats the experiment and testicular weight and testicular weight: body ratio at the end of the experiment. Data are expressed as the means ± SD; n = 6 animals for each group

Group	Initial body weight (g)	Final body weight (g)	Right testis weight (g)	Left testis weight (g)
Control	*323.16 ± 12.90*	*312.50 ± 10.01*	1.41 ± 0.02	1.43 ± 0.02
SC1	*323.33 ± 12.18*	*310.16 ± 11.53*	1.44 ± 0.07	1.46 ± 0.07
SC2	*323.16 ± 12.97*	*308.75 ± 10.14*	1.50 ± 0.03	1.48 ± 0.03
TM1	*323.16 ± 11.81*	*309.75 ± 11.65*	1.55 ± 0.04	1.54 ± 0.04
TM2	*322.83 ± 12.14*	*314.66 ± 11.66*	1.42 ± 0.03	1.41 ± 0.03
PMel	*323.33 ± 13.55*	*311.83 ± 11.96*	1.41 ± 0.03	1.43 ± 0.02
TMel	*323.66 ± 10.83*	*318.16 ± 7.14*	1.50 ± 0.03	1.51 ± 0.03
P	***NS***	***NS***	**NS**	**NS**

SC1 = Vehicle control-1; SC2 = Vehicle control-2; TM1 = Tunicamycin1; TM2 = Tunicamycin2; PMel = Profoloctic melatonin; TMel = Treatment melatonin; n = number of rats; NS = not significant

**Table 2:** Histomorphometric assay of the rat testes. Data are expressed as the means ± SD; n = 6 rats for each group

**Group**	**Stage VII–VIII** **STD [μm] **	**Stage VII–VIII** **SEH [μm]**	**Stage XII–XIV** **STD [μm]**	**Stage XII–XIV** **SEH [μm]**	**Stages XIV** **tubules (%)**
**Control**	322.41 ± 0.73^a^	79.88 ± 0.33^d^	283.59 ± 0.49^b^	93.53 ± 0.41^d^	3.25 ± 0.15
**SC1**	320.76 ± 1.24^a^	81.80 ± 0.37^c^	288.71 ± 0.92^a^	100.15 ± 0.41^a^	3.16 ± 0.23
**SC2**	322.13 ± 0.56^a^	84.75 ± 0.35^a^	277.61 ± 0.44^c^	97.74 ± 0.38^b^	3.18 ± 0.26
**TM1**	311.68 ± 0.98^c^	80.55 ± 0.30^d^	276.38 ± 0.72^c^	93.25 ± 0.34^d^	2.76 ± 0.23
**TM2**	315.84 ± 1.10^b^	83.58 ± 0.37^b^	277.68 ± 0.84^c^	95.99 ± 0.39^c^	2.91 ± 0.18
**PMel**	303.22 ± 0.55^d^	76.71 ± 0.28^e^	265.55 ± 0.76^d^	93.87 ± 0.38^d^	2.93 ± 0.16
**TMel**	311.61 ± 0.72^c^	82.72 ± 0.34^bc^	281.93 ± 0.73^b^	101.10 ± 0.39^a^	2.97 ± 0.28
**P**	***	***	***	***	**NS**

STD: Seminiferous tubule diameter, SEH: Seminiferous epithelial height, NS: not significant

a, b, c, d, e: Different superscripts in the same column indicate significant differences

*** *P*<0.001

## Discussion

Recent studies have shown ER stress to be associated with male fertility/infertility. Infertility has been reported to possibly develop due to an impaired ER stress response during the male reproductive period (33-35). Various physiological conditions, environmental stimulants, and pharmaceutical agents cause impaired ER homeostasis. This situation causes ER stress in the male reproductive system (35). Surprisingly, recent evidence has shown that oxidative stress induced by ROS production causes ER stress. The cellular antioxidant mechanism is necessary to clean and reduce ROS production to prevent cross-mixing between oxidative stress and ER stress (36). Through direct and indirect mechanisms, melatonin protects DNA and other components from damage while limiting oxidative stress in cells. While melatonin is known to indirectly increase antioxidant enzyme activity, it is also stated to increase mRNA expression through membrane receptors (37). Keeping in view this promising evidence, our study investigated the effects of prophylactic and therapeutic doses of melatonin on ER stress-induced male infertility. No earlier study has reported the prophylactic use of melatonin. Therefore, this application was included in the study. We investigated the effects of using melatonin, whose recent exogenous use has been shown to have beneficial effects against ER stress in testicular tissue. ER stress induced by TM increased GRP78 levels, oxidative stress, and apoptosis rates in testicular tissue, and administration of melatonin improved these changes. The most important finding of this study is that prophylactic administration of melatonin before cell damage occurs may be more effective in preventing ER stress. We found that, while prophylactic administration of melatonin increased antioxidant activity in the experimental groups, it decreased GRP78 staining intensity and expression levels. In addition, the apoptotic index and TWAC% values were lowered in the therapeutically administered melatonin group compared with the prophylactically administered melatonin. When compared with the tunicamycin group, the tubular structure in the melatonin groups was not different from that of the control group. To the best of our knowledge, this is the first study to investigate both prophylactic (PMel) and therapeutic (TMel) uses of melatonin against tunicamycin-induced ER stress in testicular tissues of rats.

The ER stress marker (GRP78) was immunohistochemically stained at different densities in cells in the seminiferous tubule and interstitial area. While this staining was the most intense in groups that cause ER stress through TM, we found that melatonin applications decreased this intensity. The present study also investigated the expression levels of GRP78 using western blotting. The immunohistochemistry results of GRP78 intensity from the staining are consistent with the western blotting results of GRP78 protein expression. In the present study, ER stress biomarker GRP78 levels were elevated in rats treated with TM compared with the control/untreated rats. GRP78 in tunicamycin-treated rats was attenuated by administration of melatonin. In both examinations, melatonin used as prophylactic was found to keep GRP78 expressions at the same levels as in the control groups. In testicular tissue, GRP78 is expressed in almost all cell types found in the seminiferous tubules. Strong immunohistochemical staining of GRP78 was noted in the cytoplasm surrounding the nuclei of spermatocytes and round spermatids (38, 39). A recent study using immunofluorescence staining reported GRP78 to be mainly in the cytoplasm of developing germ cells (40). As GRP78 stains the seminiferous tubular germ cells, tubular staining intensity was evaluated for GRP78 in our study. Previous studies have stated the substances that cause toxicity in the testicles increase the level of GRP78 induced by ER stress, which can be revealed by immunohistochemistry and western blot (41, 42). As a result of ER stress induced in rats by dibutyl phthalate (DBP), GRP78 was mostly localized in the cytoplasm of spermatogenic cells, and its density increased significantly (43). In addition, other studies have reported the reduction of increased GRP78 levels by administration of melatonin. A study reported that testicular GRP78 expression increased significantly because of cadmium-induced testicular ER stress in mice, and melatonin reduced the cadmium-induced increased levels of testicular GRP78 (44). Therapeutic administration of melatonin for 14 days to diabetes-induced ER stress in mice decreased the level of GRP78 expression in testicular tissue (10). Melatonin treatment attenuated ER stress by reducing the GRP78 expression resulting from ER stress in mice fed high-fat diets (42). The use of melatonin in ER stress induced by palmitic acid (PA), which causes lipotoxicity in mice, effectively blocked PA-induced ER stress in testicular tissue and maintained the ER homeostasis (45). Melatonin decreased the expression of ER stress markers such as GRP78, ATF6, and XBP1 in busulfan-induced ER stress in mouse testicular tissue (46). These results may indicate melatonin supplementation to down-regulate GRP78 expression in rat testes. Melatonin administration may serve to preserve cell viability by regulating GRP78 through the possible mechanisms of autophagy and apoptosis.

TUNEL positive reacting apoptotic cells were detected in the seminiferous tubules belonging to all groups. However, the number of apoptotic cells was found to be higher in the groups administered with TM to create ER stress compared with the other groups. While the amount of apoptosis in the TMel group was not different from that in the control groups, fewer apoptotic cells were observed in the TMel group than in the PMel group. The most apoptotic tubules were found in the tunicamycin-administered groups. In a study, the use of melatonin against PA-induced lipotoxicity in mice significantly reduced PA-induced apoptosis, while treatment with melatonin alone did not alter the proportion of apoptotic cells in testicular tissue compared with the control group (45). Melatonin reduced the apoptosis induced by busulfan in mouse testicular tissue by suppressing the expression of ER stress-related apoptotic genes (46). ER stress-mediated germ cell apoptosis induced by cadmium in testicular tissue decreased the apoptotic index and TWAC% rates with melatonin treatment (44). Increasing evidence has been found supporting the notion that ER stress and oxidative stress play important roles in the formation of cellular apoptosis. ER stress is one of the main causes of apoptosis. Disturbances in ER homeostasis cause the accumulation of misfolded proteins, activating CHOP, which is an important pro-apoptotic factor, and inducing UPR-mediated apoptosis (47). Consistent with this evidence, we believe melatonin might be able to alleviate apoptosis in seminiferous tubules by alleviating ER stress directly and indirectly in multiple pathologies. However, the molecular mechanism underlying this situation still needs to be explained.

MDA levels were higher in the tunicamycin-applied groups compared with the control groups and lowest in the PMel group. When examining SOD activity, levels were determined to be high in the melatonin-applied groups, especially in the PMel group. In addition, the lowest SOD activity was detected in the TM1 group. The oxidative state caused by ER stress in different tissues has been investigated in some studies by determining MDA and SOD levels. While MDA levels have been shown to increase in liver tissue due to ER stress induced by TM in mice, SOD activity decreases (48). Increased MDA levels and decreased SOD activity in liver tissue due to ER stress-related hepatic injury in liver tissue using TM in mice are regulated by applying resveratrol (49). Quercitin used in ER stress-mediated oxidative stress induced by TM in the endothelial cell decreases MDA levels and increases SOD activity (50). Increased MDA levels and decreased SOD activity due to di (2-ethylhexyl) phthalate-induced testicular injury in adult mice were improved by administrating melatonin (51). The use of melatonin against cadmium toxicity in mice improved the cadmium-induced MDA accumulation and SOD levels (52). Not many studies are found regarding the effects of exogenous melatonin administration on MDA and SOD parameters resulting from ER stress. The results of our study show that exogenous administration of melatonin improves oxidative stress induced by the accumulation of ROS products and thus ER stress. 

Degenerative changes were more pronounced in the tunicamycin-administered groups than in the melatonin-applied groups. One day after TM injection, the TM1 group had seminiferous tubular diameter shrinkage, apoptotic cells, sub-basal vacuolization, and sporadic germ cell shedding. Remarkably, many apoptotic cells were found in the TM2 group with larger and more numerous vacuolization. The melatonin-applied groups’ tubular structures resembled those in the control group but with a decrease in tubular diameter. Degenerative/atrophic indicators were observed in some tubules in the PMel group, with fewer apoptotic cells being detected compared with the tunicamycin groups. While epithelial vacuolization was found in the TMel group, no apoptotic cells were found. In a study, after injecting 200 μg/kg and 300 μg/kg TM into the rats, damage began in the seminiferous tubular epithelium on the 3rd and 5th days, with much more damage occurring after the 19th day (53). By applying melatonin to rats for 21 days, spermatogenic cells were separated from the seminiferous tubule, and atrophic areas were observed in some parts of the testis in addition to normal histological seminiferous tubules (54). When investigating the therapeutic effect of melatonin against gentamicin-induced testicular toxicity in rats, the control and melatonin groups had normal testicular structures (55). Considering that the damage TM induces in testicular tissue is caused by local ischemia, the anti-inflammatory and antioxidant effects as well as the anti-ischemic effect of melatonin can prevent this damage. Thereby, the tubular appearance similarities with the control group may be a possible result of melatonin usage.

The diameter of the stage VII-VIII seminiferous tubules in the experiment groups decreased compared with the control groups. The lowest stage VII-VIII seminiferous tubular diameter and epithelial height values were found in the PMel group. When comparing the groups’ stage XII-XIV seminiferous tubular diameters, the largest diameter was noted in the control group and the lowest in the PMel group. This study found both TM and melatonin to decrease stage VII-VIII seminiferous tubular diameters. Studies have reported that melatonin is used for different purposes in rats and mice, and it reduces the tubule diameter in the testicle (54, 56-58). The application of exogenous melatonin is suggested to cause a decrease in the testicular tissue size of rats. Although the mechanism underlying this has yet to be fully explained, it is thought that melatonin receptors in the testicular tissue do affect the secretion of GnRH and testosterone, causing shrinkage in the testicles and tubules (59). The results of our study showed a decrease in the diameter of seminiferous tubules where TM and melatonin had been applied. The endocrine function and spermatogenesis can be thought to have been negatively affected due to the decrease in tubular diameter, the underlying mechanism of which is not fully explained.

Since no significant difference was observed between the melatonin-administered groups and the control groups in any respect, such a group was not included in the present study. In future studies on this subject, investigating proteins associated with UFR such as IRE1, ATF6, and XBP-1, markers such as CHOP and caspase 3 that play a role in ER stress-related apoptosis, and testosterone level and sperm examination will contribute to our understanding of this issue better along with elimination of missing aspects of this issue.

## Conclusion

Based on our data, administering melatonin before exposure to toxic substances and agents is more effective than the therapeutic use of melatonin to improve cell damage and protect cells from ER stress, cellular apoptosis, and oxidative stress. The prophylactic effect of melatonin may be due to the powerful antioxidant and free radical scavenger effects of melatonin. Further investigations are needed to evaluate the mechanisms by which melatonin may protect testicular tissue against ER stress.

## Authors’ Contributions

MT and  UE conceived the study and design, analyzed the data, prepared the draft manuscript, critically revised the paper, supervised the research, and approval the final version to be published.  

## Conflicts of Interest

The authors declare that they have no conflicts of interest.
